# Diffuse B cell large ileal lymphoma presenting with obstruction and associated with Hashimoto’s thyroiditis

**DOI:** 10.1093/jscr/rjaf253

**Published:** 2025-04-28

**Authors:** Shumarova Svetlana, Koleva Liliya, Karamisheva Vesela

**Affiliations:** Department of Surgery, University Hospital “Aleksandrovska” Sofia, Bulgaria, Medical University, Sofia, Bulgaria; Department of Gynecology, Faculty of Medicine, Medical University of Sofia Bulgaria, UMBALSM “Pirogov”, Sofia, Bulgaria; Department of Obstetrics and Gynecology, Faculty of Medicine, Medical University of Sofia Bulgaria, SBALAG “Maichin dom”, Sofia, Bulgaria

**Keywords:** diffuse B cell large lymphoma, small bowel lymphoma, ileum, obstruction

## Abstract

Primary small intestinal B cell lymphoma is a rare and often presents in its complicated form with the presence of perforation, obstruction, or bleeding. We present a 74-year-old woman with nonspecific complaints of persistent constipation. Ultrasound and computed tomography revealed a tumor of the ileum with obstruction and dilation of the intestine. The patient underwent small bowel resection with total hysterectomy. A suspected etiological factor with oncological potential in her is the long-term use of levothyroxine for Hashimoto’s thyroiditis.

## Introduction

Non-Hodgkin lymphoma is a malignant tumor originating from the submucosal lymphoid tissue. The most common extranodal location is the gastrointestinal tract, and it can develop along its entire length, with the most common locations being the stomach (51.3%), small intestine (34.6%), ileocecal region (9%), and rectum and colon (5.1%) [1]. The predominant form of non-Hodgkin lymphoma is B-cell lymphoma (87.5%), while T-cell lymphoma accounts for about 12.5% [1]. There are several types of B-cell lymphoma, with diffuse large B-cell lymphoma prevailing (85.8%) over the others (MALT lymphoma, mantle cell lymphoma, follicular lymphoma, B- lymphoblastic lymphoma) [1].

The clinical symptoms of small bowel lymphomas are usually nonspecific, and in the presence of symptoms such as abdominal pain, weight loss, night sweats, constipation, they suggest an advanced disease. Often the first substantial clinical manifestations of the small intestine form are bleeding [2, 3], ileus [2, 4] or perforation [2, 5], which requires urgent surgical intervention. The ~5-year overall survival rate (5-YSR) is 48.5% and 5-YSR is similar regardless of the type of primary treatment (chemotherapy alone vs. surgery/chemotherapy, 50.7% vs. 45.3%, *P* = 0.582) [2]. We present a 74 year-old woman with established B cell large ileal lymphoma with clinical signs of obstruction and associated with a long- standing history of Hashimoto’s thyroiditis.

## Clinical case

**Figure 1:**
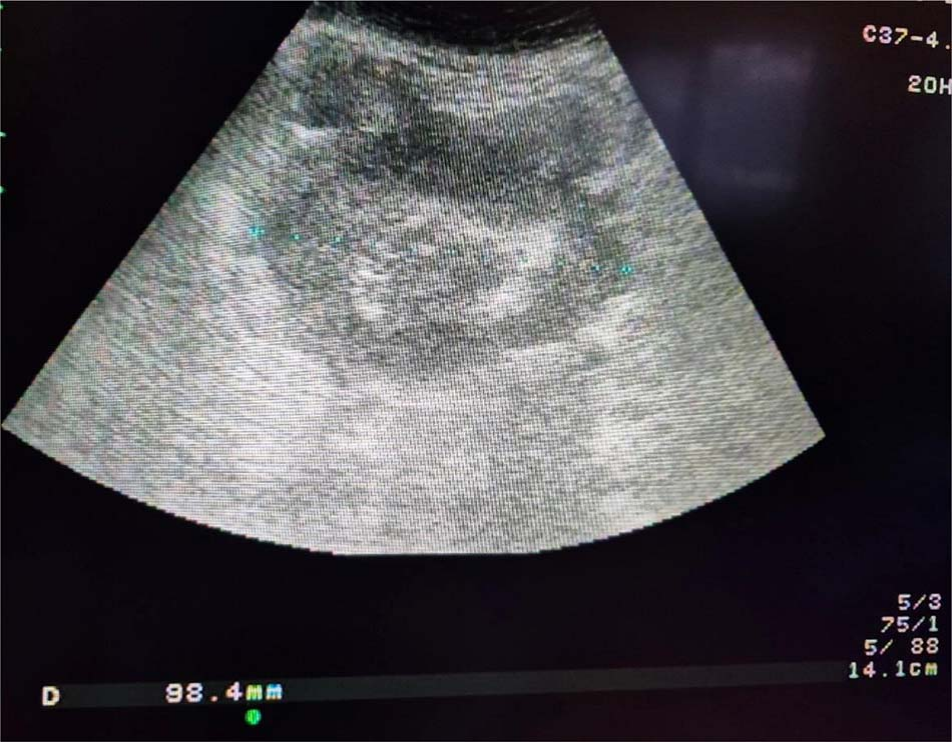
Ultrasound image of the small intestinal tumor.

**Figure 2:**
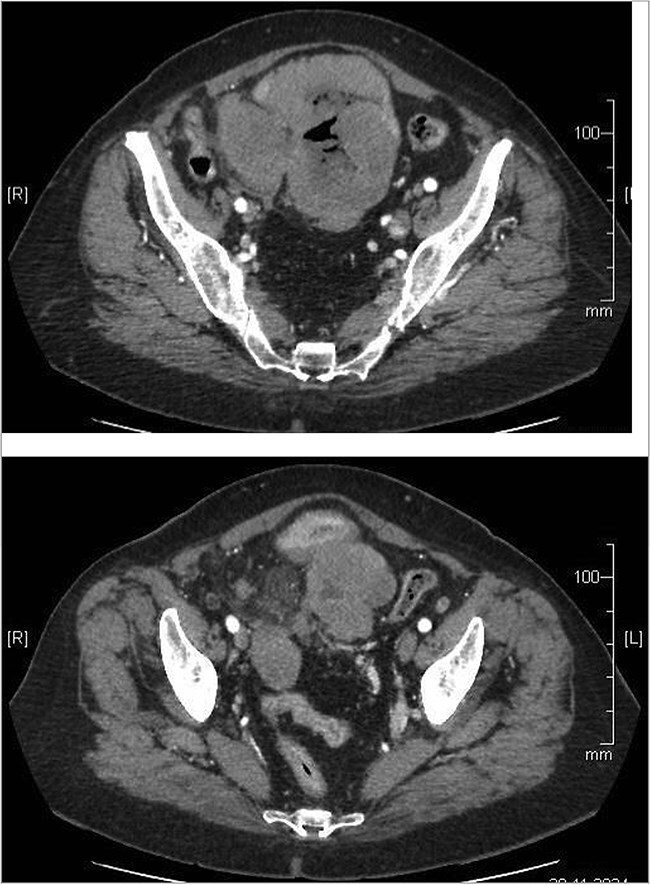
Abdominal CT showing small bowel tumor and similar to both ovaries.

A 74-year-old woman complains of severe constipation, difficult to respond to laxatives and abdominal pain, nausea. A primary abdominal ultrasound (US) examination revealed a hypoechoic tumor formation in the lower abdomen, with uneven outlines and hyperechoic zones in it, with dimensions of ~10 cm/day (Fig. 1). The patient was referred for a computed tomography (CT) scan of the abdomen, which revealed a soft tissue formation with axial dimensions of 130/93 mm heterogeneous structure and infiltrative growth, affecting a segment of the ileum and an adjacent dilated small intestinal loop (Fig. 2). Secondary dissemination along the peritoneum with many soft tissue formations of different sizes formed in the abdomen and small pelvis. Identical lesions are described in the structures of both ovaries bilaterally, the larger of which is 47/34 mm/day. Laboratory parameters revealed anemia with values of Hg-103 g/L, and low values of total protein-60 g/L. The patient has a history of Hashimoto’s thyroiditis and is on replacement therapy with L-thyroxine. During hospitalization, she showed good compensation with a slightly increased TSH- 4.88 mU/L (normal 0.27–4.2) and normal FT4–17.7 pmol/L (normal 12–22). Tumor markers CEA, CA 125, and CA 19-9 were not elevated.

**Figure 3:**
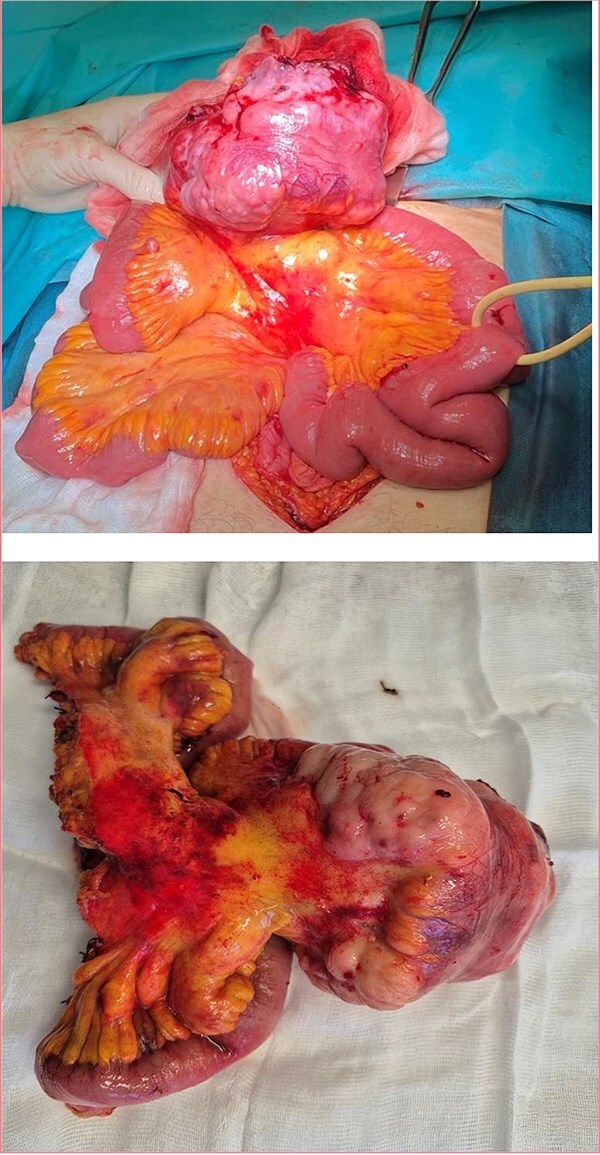
Ileal tumor obturating the intestine and visibly enlarged mesenteric lymph nodes.

**Figure 4:**
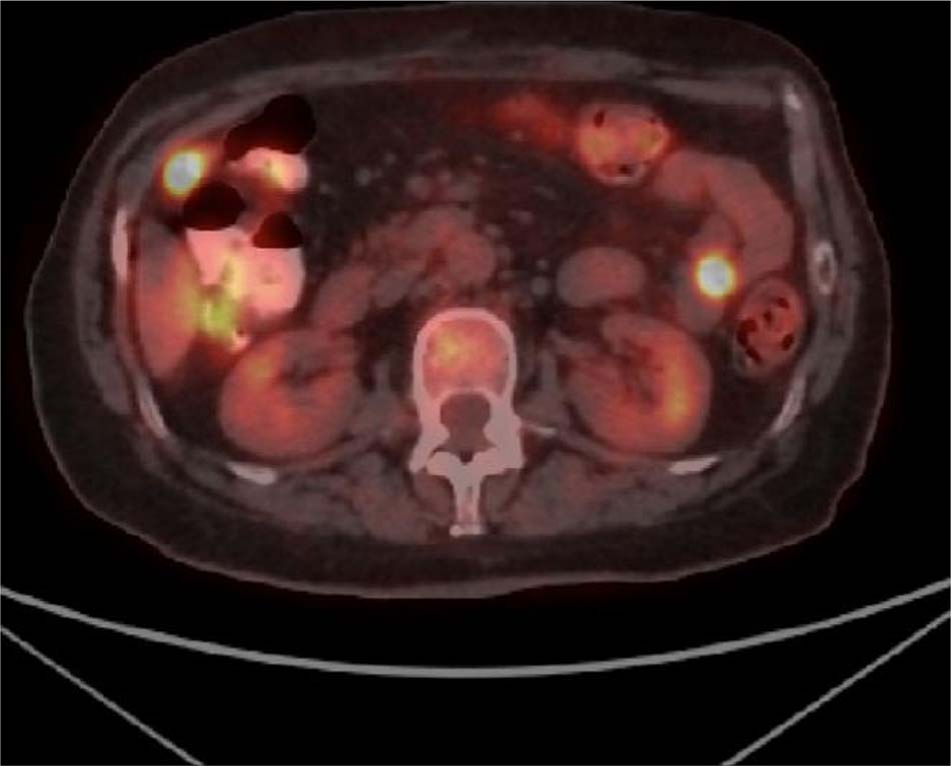
PET CT showing metabolic activity in abdominal and pelvic lymph nodes.

Taking into account clinical and imaging data for obstruction of the small intestine by the tumor and the lack of other options for histological analysis, a decision was made for laparotomy. An ileal tumor measuring ~13 cm/day with necrotic changes, involving the entire small intestine loop with dilatation of the proximal part of the small intestine, and multiple enlarged lymph nodes near the tumor were found (Fig. 3). The tumor also involved the anterior abdominal wall, separately similar lesions were visualized in both ovaries with dimensions of ~5 cm/day. The small intestine was resected with subsequent anastomosis and a total hysterectomy with bilateral salpingo-oophorectomy was performed. The postoperative period was trouble- free and the patient was discharged on the fifth postoperative day, and scheduled for systematic chemotherapy. Positron emission tomography (PET CT) showed the presence of peritoneal lesions, abdominal and pelvic lymph nodes (Fig. 4). Histological analysis established diffuse large B-cell lymphoma (DLBCL), GC subtype involving transmural small intestine, mesenteric lymph nodes, mesovarium, peritoneum. Metastatic nodules adhering to both ovaries. Immunohistochemical study revealed diffuse strong expression of CD 10 and CD 20 in tumor cells, Ki-67- over 90% proliferative activity; MUM1: negative reaction; BCL-6: positive nuclear expression in about 70% of the neoplastic population; CD3,CD5: positive stromal T cells; CD23: membrane expression in small groups of cells at the invasion front; Cyclin D1: lack of expression in tumor cells under positive internal control.

## Discussion

Primary small bowel lymphoma is rare but extremely challenging to diagnose, firstly due to the non-specific symptoms of patients, secondary due to the difficult availability of material for histological analysis. This neoplasm mainly affects age groups with an average age of over 57 years according to some authors [2, 6] with male prevalence between 1.86:1 and 2.1:1 [2, 6]. Diffuse B cell lymphoma may result from genetic mutations affecting protooncogenes and tumor suppressor genes [7], and genetic changes in the BCL6 gene may be observed in 20% to 40% of patients [7]. In our case, there is a positive nuclear expression in 70% of the neoplastic population. The etiological factors for its appearance can be very diverse: immunosuppressive therapy; transformation from different types of lymphomas; family or personal history of lymphoma, radiation, chemotherapy; obesity; history of autoimmune diseases [7]. A risk factor in our case is the long-term intake of L-thyroxine due to Hashimoto’s thyroiditis, and evidence of this is pointed in other sources [8]. A large recent analysis performed by Wu *et al.* [9] showed that 50% of levothyroxine users had a higher risk of cancer of any nature (AOR: 1.50, 95% CI:1.46–1.54; *P* < 0.0001), compared to non-users. This should be taken into account in all patients using levothyroxine, i.e. they should be considered at risk of cancer development, and should be screened frequently and purposefully.

There is no defined treatment algorithm in patients with small intestine B cell lymphoma. A large proportion of patients are asymptomatic or have non-specific symptoms, and often the first manifestation of treatment is a surgical emergency due to complications such as bleeding, obstruction or perforation. Currently, the combination of CHOP chemotherapy and rituximab is the main treatment for small intestine diffuse large B cell lymphoma [1]. According to Cha *et al.* [2], the approximate 5-YSR is 48.9%. They compared survival according to the primary site of involvement and found a 5-YSR of 32.5% (*P* = 0.027), 64.3% (reference), 46.5% (*P* = 0.113), and 49.8% (*P* = 0.024) for small intestine, ileocecal region, colon, and multiple sites, respectively. With a median follow-up time of 72 months of patients with primary intestinal DLBCL, Chen *et al.* [6] reported OS at 1 year, 3 year, and 5 year, respectively, 79.55%, 45.45%, and 28.41%.

## Conclusion

Due to nonspecific clinical symptoms, primary small intestine DLBCL are usually detected at an advanced stage, which worsens overall survival. It is necessary to associate certain risk factors with the future development of oncological disease in order to prevent or detect the disease at an early stage. Therefore, new risk evaluation protocols need to be created in order to insure the timely screening and early diagnostics.
